# Patient acceptable symptom state and treatment failure threshold values for work productivity and activity Impairment and EQ-5D-5L in osteoarthritis

**DOI:** 10.1007/s11136-024-03602-6

**Published:** 2024-02-26

**Authors:** Ali Kiadaliri, Anna Cronström, Leif E. Dahlberg, L. Stefan Lohmander

**Affiliations:** 1 Department of Clinical Sciences Lund, Orthopedics, Lund University, Lund, Sweden; 2 Arthro Therapeutics, Malmö, Sweden; 3 Department of Health Sciences, Lund University, Lund, Sweden; 4 Department of Community Medicine and Rehabilitation, Umeå University, Umeå, Sweden; 5 Clinical Epidemiology Unit, Skåne University Hospital, Remissgatan 4, 221 85 Lund, Sweden

**Keywords:** EQ-5D-5L, Work productivity and activity impairment, Patient acceptable symptom state, Osteoarthritis, Digital treatment

## Abstract

**Objective:**

To estimate patient acceptable symptom state (PASS) and treatment failure (TF) threshold values for Work Productivity and Activity Impairment (WPAI) measure and EQ-5D-5L among people with hip or knee osteoarthritis (OA) 3 and 12 months following participation in a digital self-management intervention (Joint Academy®).

**Methods:**

Among the participants, we computed work and activity impairments scores (both 0–100, with a higher value reflecting higher impairment) and the Swedish hypothetical- (range: − 0.314 to 1) and experience-based (range: 0.243–0.976) EQ-5D-5L index scores (a higher score indicates better health status) at 3- (*n* = 14,607) and 12-month (*n* = 2707) follow-ups. Threshold values for PASS and TF were calculated using anchor-based adjusted predictive modeling. We also explored the baseline dependency of threshold values according to pain severity at baseline.

**Results:**

Around 42.0% and 48.3% of the participants rated their current state as acceptable, while 4.2% and 2.8% considered the treatment had failed at 3 and 12 months, respectively. The 3-month PASS/TF thresholds were 16/29 (work impairment), 26/50 (activity impairment), 0.92/0.77 (hypothetical EQ-5D-5L), and 0.87/0.77 (the experience-based EQ-5D-5L). The thresholds at 12 months were generally comparable to those estimated at 3 months. There were baseline dependencies in PASS/TF thresholds with participants with more severe baseline pain considering poorer (more severe) level of WPAI/EQ-5D-5L as satisfactory.

**Conclusion:**

PASS and TF threshold values for WPAI and EQ-5D-5L might be useful for meaningful interpretation of these measures among people with OA. The observed baseline dependency of estimated thresholds limits their generalizability and values should be applied with great caution in other settings/populations.

**Supplementary Information:**

The online version contains supplementary material available at 10.1007/s11136-024-03602-6.

## Introduction

Patient-reported outcome measures (PROMs) have increasingly been advocated to assess treatment effect from patient’s perspective in the clinical setting [1]. However, interpreting and communicating numeric PROMs values in a clinically relevant manner can be challenging since these values may not correlate with a patient’s perceived improvement and well-being [2, 3]. To address this, minimal clinically important difference (MCID)–defined as the smallest change in PROM scores that patients perceive as beneficial–was introduced [4]. While MCID measures how much improvement is needed for patients to feel “better,” it doesn’t provide insights on whether patients are satisfied (feel “good”) about their current status [5]. In other words, a meaningful improvement in a PROM does not necessarily reflect a desirable state, especially if a patient was in a “terrible” state to begin with [6]. In response, concepts of “patient acceptable symptom state” (PASS) [7] and “treatment failure” (TF) [8] have been introduced. PASS is the threshold above which patients will consider themselves “well” and satisfied with treatment [5], while TF is the threshold below which patients consider their symptoms to be unsatisfactory to a degree that they consider the treatment has failed [8].

While previous studies established PASS [2, 3, 7, 9–12] and TF [2, 10, 12] thresholds for PROMs assessing symptoms among patients with osteoarthritis (OA), less attention has been paid to generic PROMs measuring general health-related quality of life (HRQoL). Work Productivity and Activity Impairment: Specific Health Problem (WPAI:SHP) and EQ-5D-5L are two PROMs that are advocated for assessing HRQoL, including work and activity limitations in OA [13, 14]. A recent study reported that across five common instruments to measure work impairment, WPAI was the instrument preferred by participants [13]. EQ-5D is a simple self-administered questionnaire which is commonly used in the OA context and is the most commonly collected PROM in the Swedish National Quality Registers [15]. Although a few studies estimated the PASS thresholds for EQ-5D-3L in OA [16–19], to our knowledge, only one recent study has estimated this for EQ-5D-5L in OA [20] and there is no reported PASS threshold for WPAI in OA or any other condition. Furthermore, while patient education, self-management, and exercises are recommended as core first-line treatments for all persons with OA, all previous (EQ-5D) PASS thresholds were estimated among people undergoing surgical treatment which is recommended as the last resort for a minority of people with severe signs and symptoms of OA [21]. This implies that current PASS thresholds might not be applicable for general OA population. More importantly, to our knowledge, no previous study has reported TF thresholds for either EQ-5D or WPAI in any population. Combination of PASS and TF thresholds can aid to determine the scores representing an acceptable or failed post-treatment outcome. To facilitate meaningful interpretation of the values reported for EQ-5D-5L and WPAI, the present study aimed to establish PASS and TF thresholds for these PROMs among participants of a digital first-line self-management program for OA.

## Methods

This is a secondary analysis of register data obtained from consecutive participants of a digitally delivered self-management program for hip and knee OA, known as Joint Academy®, described in details elsewhere [22, 23]. In short, inspired by the Swedish first-line face-to-face management program for OA (known as “Better management of patients with OsteoArthritis” which exists as a National Quality Register), the digital program was introduced in Sweden in 2016 and is targeted toward exercise, physical activity, and education delivered by a smartphone application. It contains video lectures on OA, physical activity, and self-management as well as individualized exercises and a possibility to chat asynchronously with a physical therapist during the treatment. The program is covered by the national healthcare system in Sweden.

### Participants

All participants aged 20 years and older with self-reported doctor/physiotherapist diagnosed hip or knee OA enrolled in the digital program between January 1st, 2019 and September 30th, 2021, who provided informed consent for research at enrollment were eligible for the current study (*n* = 16,640). Of these, we excluded those with missing responses to anchor questions at both follow-ups. We extracted the data in January 2022.

### PROMs

The EQ-5D-5L is a generic preferences-based health measure consisting of the five dimensions of mobility, self-care, usual activities, pain/discomfort, and anxiety/depression. Each dimension has five levels of severity: no problems, slight problems, moderate problems, severe problems, and unable to /extreme problems, resulting in 3125 (5^5) unique health states [24]. The responses to the EQ-5D-5L can be summarized as a single score anchored at 1 (full health) and 0 (a state equivalent to dead) using a reference value set [24]. Values less than 0 are possible representing health states considered to be worse than dead. We used the Swedish hypothetical-based [25] and experienced-based [26] value sets to compute the EQ-5D-5L index score. The hypothetical-based value set ranges from -0.314 (worst health state) to 1 (best health state), while the experienced-based value set ranges from 0.243 to 0.976. We used both value sets to assess the potential differences between experience- and hypothetical-based scores.

The WPAI:SHP is a six-item validated instrument to measure the impact of a person’s specific health problem (OA in the current study) on work and daily non-work-related activities during the past 7 days [27]. Work impairment is calculated as summation of absenteeism + presenteeism. *Absenteeism* measures the percent work time missed due to OA and is calculated as [hours missed due to OA/ (hours missed due to OA + hours actually worked)]. *Presenteeism* measures the extent to which OA affected productivity while working. This was estimated by multiplying the percent actually working by the extent of work impairment due to OA (11-point numerical rating scale [NRS], 0 = OA had no effect on my work and 10 = OA completely prevented me from working). Activity impairment measures the extent to which OA influenced the ability to do regular daily activities (11-point NRS, 0 = OA had no effect on my daily activities and 10 = OA completely prevented me from doing my daily activities). Both work and activity impairments are expressed as percentages with higher numbers indicating greater impairments [27]. We measured work impairment only among the participants aged 70 years and younger who were employed when responding to the questionnaire.

### Anchor questions

We evaluated PASS at 3 and 12 months after enrollment in the digital program by asking the question: “Considering your knee/hip function, do you feel that your current state is satisfactory? With knee/hip function, you should take into account all activities during your daily life, sport and recreational activities, your level of pain and other symptoms, and also your knee/hip-related quality of life.” The response options were “yes” or “no” [8]. We then asked the participants who answered “no” to the PASS anchor question to answer a second question related to TF: “Would you consider your current function as being so unsatisfactory that you think the treatment has failed?” (yes/no) [8].

### Data analysis

Patient characteristics at enrollment are reported as mean (standard deviation [SD]) for continuous variables and number/proportions for categorical variables. We computed standardized mean difference to compare baseline characteristics of participants included and excluded from the analyses and applied a threshold of 0.1 to define important difference [28]. We used standardized mean difference instead of t tests or other statistical tests of hypothesis because it is not influenced by sample size and allows for comparison of the relative balance of variables measured in different units [28].

Using the responses to the PASS and TF anchor questions, we created a variable with 3 categories: (1) participants with a satisfactory symptom state (PASS = yes), (2) participants who considered the treatment failed (PASS = no & TF = yes), and (3) participants with neither an acceptable symptom state nor treatment failure (PASS = no and TF = no). We explored the distribution of PROMs across these categories. We evaluated the strength of correlations between the PROMs and this combined PASS and TF variable at each time point using Spearman’s correlation coefficient.

To estimate the PASS and TF thresholds for each PROM in each follow-up, we used an anchor-based approach known as “predictive modeling” which has been proposed to yield more precise estimates than receiver operating characteristic (ROC) approach [29]. The predictive modeling approach is based on a logistic regression, using the PASS/TF anchor responses as the dependent variable and PROMs as the single predictor:

$${\text{log}}(\frac{p}{1-p})=\alpha +\beta *PROM,$$

where p represents the proportion of satisfied people (i.e., PASS = yes/TF = no in estimating the PASS/TF thresholds for EQ-5D-5L scores where a higher score reflects better outcome and PASS = no/TF = yes in estimating the PASS/TF thresholds for WPAI:SPH where a higher score reflects a worse outcome). All people in the three categories mentioned above were included in the analysis. In estimating the PASS threshold, we treated individuals in category 1 as “yes” response and other two groups as “no” response, while in estimating the TF threshold, people in groups 1 and 3 were considered as “no treatment failure” and those in group 2 as “treatment failure.” The threshold is defined as the PROM score that corresponds to a likelihood ratio of 1. With a likelihood ratio of 1, the post-test odds of “yes” response are the same as the pre-test odds of “yes” response. However, both ROC and predictive modeling approaches may be biased if the dependent variable is unequally distributed, that is, the proportion of respondents having a satisfactory symptom state differs from 50% [30]. We therefore applied an adjustment recommended by Terluin et al. [30]. We used bootstrap replications (*n* = 1000) to obtain the threshold values (as the mean of bootstrap replications) and corresponding 95% confidence intervals (CI).

We also explored the baseline dependency of the PASS/TF thresholds. To avoid possible spurious baseline dependency, it is recommended to use a different PROM that is correlated with the PROM of interest to assess the baseline dependency [31]. Therefore, we used 11-point numerical rating scale (NRS) pain measuring pain during the last week in the joint of interest ranging from 0 (indicated no pain) to 10 (indicating the worst possible pain). We used the NRS pain median scores at enrollment to split the sample into high and low pain intensity. We conducted a subgroup analysis by osteoarthritis site (i.e., knee and hip OA) and another one by age in which we divided the participants into two groups by the median age in our sample (≤ 65 years vs. > 65 years). Since work impairment is less relevant for people aged > 65 years, we did not estimate the threshold values for work impairment in our subgroup analysis by age. We employed bootstrapping (*n* = 1000) to generate 95% CI around the differences in the PASS/TF thresholds between these subgroups. In a sensitivity analysis, we estimated the thresholds only among participants with responses in both follow-ups (complete case analysis). Statistical analyses were implemented in RStudio (version 2022.07.2) and Stata v.17.

## Results

Of 16,640 eligible participants, we excluded 2007 (12.1%) individuals who did not respond to anchor questions at any follow-up. There were differences in the baseline characteristics of participants included and those excluded, with the latter being older, with a higher proportion of hip OA and having poorer PROMs scores than those included (Table 1). A total of 14,633 individual aged 24–94 years with mean (SD) age 64.1 (9.1) years and 75.5% females were included. Of included participants, 14,607 and 2707 provided 3- and 12-month responses, respectively. It should be noted that the smaller sample size at 12-month follow-up was mainly due to the study time-frame. That is, most participants didn’t reach their 12-month follow-up when data were extracted in January 2022 (e.g., 9199 individuals enrolled between February and September 2021). For participants included in the study, the mean (SD) hypothetical and experience-based EQ-5D-5L index scores were 0.84 (0.17) and 0.82 (0.11) at baseline, respectively. The corresponding figures for WPAI–work and –activity impairments were 24.3 (24.8) and 39.3 (23.7). The correlation coefficients between PROMs and anchor questions were generally ≥ 0.35 with higher values at 12- than 3-month follow-up (Table 2).

**Table 1:** Baseline characteristics of persons enrolled in the digital program

Variable	Included			Excluded	Standardized mean difference^b^
	3 months	12 months	All		
N	14,607	2707	14,633	2007	–
Female, *n* (%)	11,028 (75.5)	2061 (76.1)	11,045 (75.5)	1516 (75.5)	− 0.001
Age, mean (± SD)	64.1 (9.1)	64.3 (8.6)	64.1 (9.1)	65.6 (10.3)	− 0.164
Body mass index, mean (± SD)	27.2 (4.7)	27.0 (4.8)	27.2 (4.7)	27.2 (4.8)	− 0.003
NRS Pain, mean (± SD)	5.1 (1.9)	5.0 (1.9)	5.1 (1.9)	5.2 (2.1)	− 0.046
Hypothetical-based EQ-5D-5L score, mean (± SD)	0.84 (0.17)	0.85 (0.17)	0.84 (0.17)	0.81 (0.20)	0.166
Experience-based EQ-5D-5L score, mean (± SD)	0.82 (0.11)	0.82 (0.11)	0.82 (0.11)	0.81 (0.13)	0.149
WPAI–overall work impairment (%), mean (± SD)^a^	24.3 (24.8)	24.3 (24.5)	24.3 (24.8)	26.1 (25.7)	− 0.073
WPAI–activity impairment (%), mean (± SD)	39.3 (23.7)	39.4 (23.5)	39.3 (23.7)	41.8 (24.9)	− 0.101
Education, *n* (%)					
Less than high school	11,180 (8.1)	184 (6.8)	1181 (8.1)	183 (9.1)	− 0.037
High school	5244 (35.9)	880 (32.5)	5252 (35.9)	711 (35.4)	0.010
College/university	8183 (56.0)	1643 (60.7)	8200 (56.0)	1113 (55.5)	0.012
Knee as the index joint, *n* (%)	8756 (59.9)	1677 (62.0)	8771 (59.9)	1067 (53.2)	0.137

NRS Pain = 0–10 (higher value indicates more pain), Work/Activity impairment = 0–100 (higher value indicates higher impairment)

*SD* standard deviation, *NRS* numeric rating scale, *WPAI* work productivity and activity impairment

^a^For employed participants aged 70 years and younger (*n* = 5464 for all, *n* = 5453 for 3 months, *n* = 967 for 12 months, and *n* = 619 for excluded)

^b^All included vs. excluded with a value < 0.10 suggesting comparable characteristics

**Table 2:** Spearman correlation coefficients between anchor questions and patent-reported outcome measures

Measure	3-month	12-month
Hypothetical-based EQ-5D-5L score	− 0.47	− 0.52
Experience-based EQ-5D-5L score	− 0.47	− 0.52
WPAI–overall work impairment	0.21	0.36
WPAI–activity impairment	0.50	0.54

**Fig. 1:**
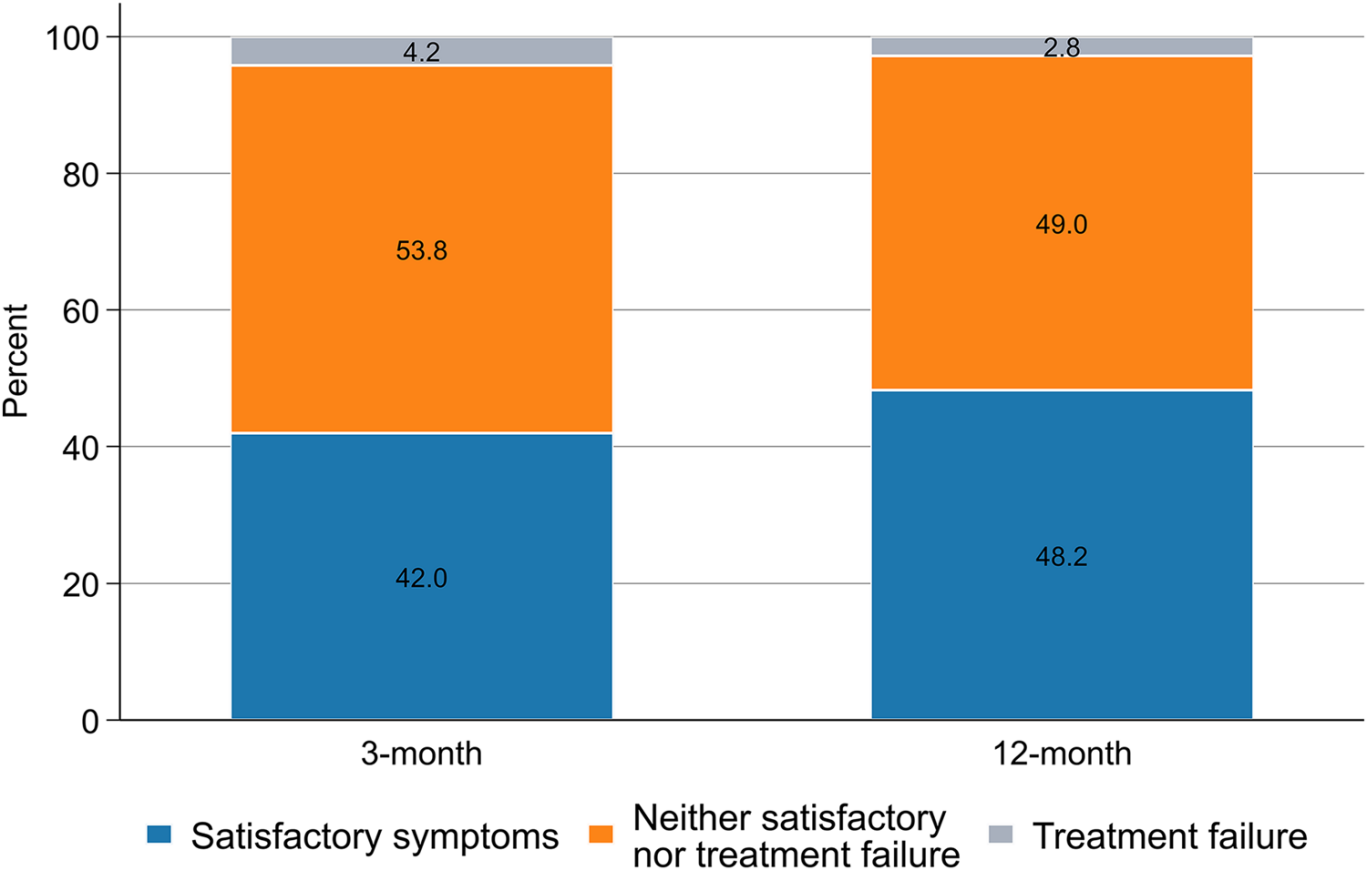
Proportions of participants who considered their symptoms satisfactory, considered the treatment had failed, or neither at 3- and 12-month follow-ups

**Fig. 2:**
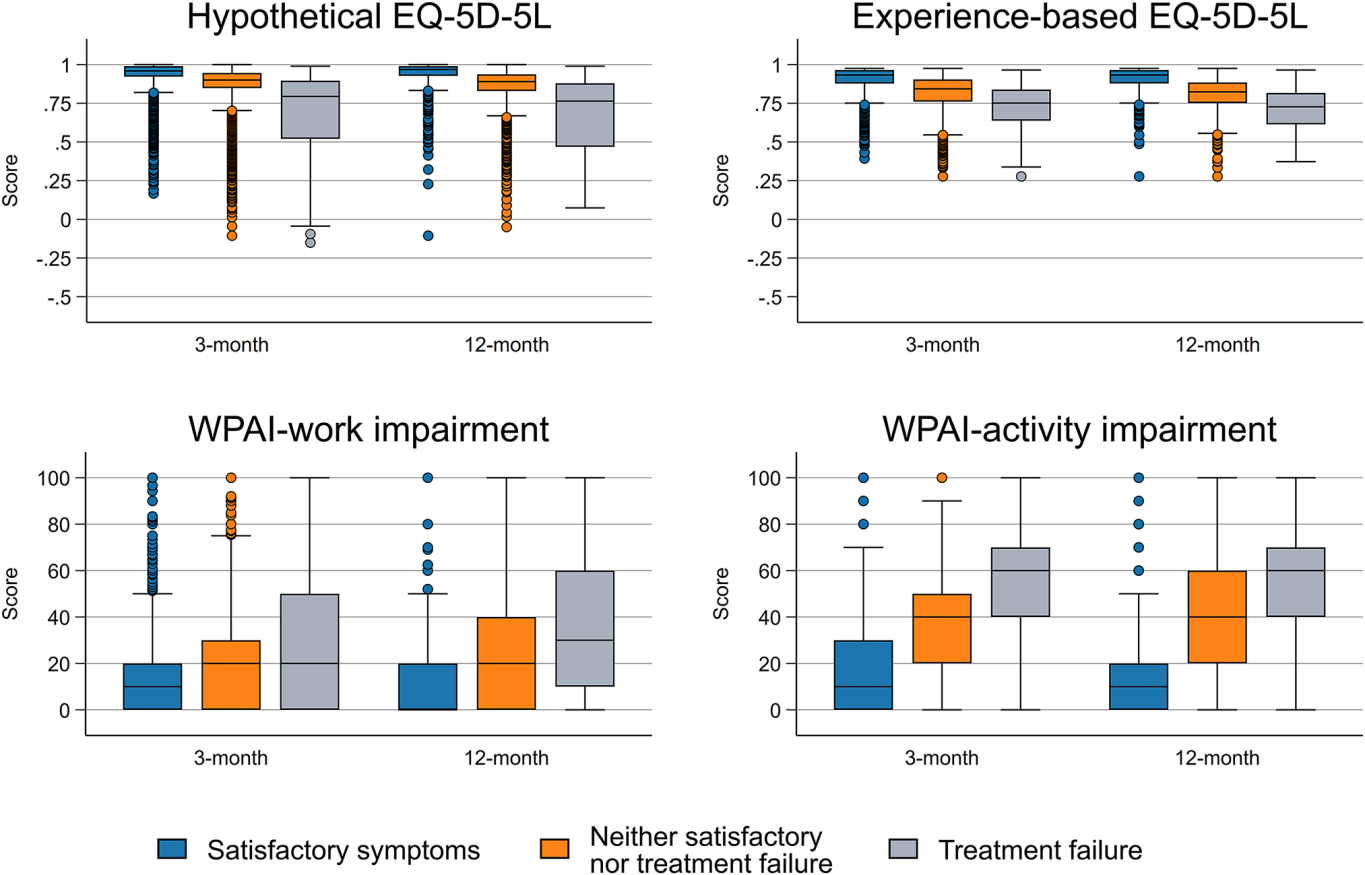
Distributions of EQ-5D-5L and Work Productivity and Activity Impairment (WPAI) scores according to patient acceptable symptom state (PASS) and treatment failure (TF) status at 3- and 12-month follow-ups

At 3-month follow-up, 6128 (42.0%) participants reported their current state as satisfactory, while 613 (4.2%) considered the treatment had failed (Fig. 1). Corresponding proportions at 12-month follow-up were 48.2% and 2.8%, respectively. Participants with a satisfactory symptom state reported better PROMs scores (i.e., higher EQ-5D-5L and lower work/activity impairments) than others in both follow-ups (Fig. 2). The PASS thresholds for the hypothetical EQ-5D-5L were 0.92 (95% CI 0.91, 0.92) and 0.91 (0.91, 0.921) at 3- and 12-month follow-ups (Table 3). Corresponding figures were 0.87 (95% CI 0.87, 0.87) and 0.87 (0.86, 0.87) for the experience-based EQ-5D-5L at these time points. The TF thresholds for hypothetical/experience-based EQ-5D-5L were 0.77 (95% CI 0.76, 0.78)/0.77 (0.76, 0.77) at 3-month and 0.75 (0.73, 0.77)/0.75 (0.73, 0.77) at 12-month follow-ups (Table 3). The PASS thresholds for WPAI–work and WPAI–activity impairments were 16 (95% CI 15, 16) and 26 (26, 26), respectively, at 3-month follow-up (Table 4). Similar PASS thresholds were estimated at 12-month follow-up. The TF thresholds for work impairment were 29 (95% CI 28, 31) and 33 (29, 39) at 3- and 12-month follow-ups, respectively. Corresponding figures for activity impairment were 50 (95% CI 49, 51) and 49 (46, 52), respectively (Table 4).

**Table 3:** Patient acceptable symptom state (PASS) and treatment failure (TF) thresholds (95% confidence intervals) for EQ-5D-5L at 3 and 12 months after enrollment in the digital program, stratified by baseline pain intensity

	All	Mild baseline pain	Severe baseline pain	Difference
*3-month responses*	*N* = 14,607	*N* = 5535	*N* = 9072	
PASS				
Hypothetical-based EQ-5D-5L score	0.92 (0.91, 0.92)	0.94 (0.94, 0.94)	0.90 (0.90, 0.91)	0.04 (0.03, 0.04)
Experience-based EQ-5D-5L score	0.87 (0.87, 0.87)	0.90 (0.90, 0.90)	0.86 (0.86, 0.86)	0.04 (0.04, 0.04)
TF				
Hypothetical-based EQ-5D-5L score	0.77 (0.76, 0.78)	0.88 (0.87, 0.89)	0.73 (0.72, 0.74)	0.15 (0.14, 0.17)
Experience-based EQ-5D-5L score	0.77 (0.76, 0.77)	0.84 (0.83, 0.84)	0.74 (0.73, 0.75)	0.10 (0.09, 0.11)
*12-month responses*	*N* = 2707	*N* = 1101	*N* = 1606	
PASS				
Hypothetical-based EQ-5D-5L score	0.91 (0.91, 0.92)	0.93 (0.92, 0.93)	0.90 (0.89, 0.91)	0.03 (0.02, 0.04)
Experience-based EQ-5D-5L score	0.87 (0.86, 0.87)	0.89 (0.88, 0.89)	0.86 (0.85, 0.86)	0.03 (0.03, 0.04)
TF				
Hypothetical-based EQ-5D-5L score	0.75 (0.73, 0.77)	0.85 (0.82, 0.87)	0.70 (0.67, 0.73)	0.15 (0.11, 0.19)
Experience-based EQ-5D-5L score	0.75 (0.73, 0.77)	0.81 (0.80, 0.83)	0.72 (0.70, 0.74)	0.10 (0.07, 0.12)

**Table 4:** Patient acceptable symptom state (PASS) and treatment failure (TF) thresholds (95% confidence intervals) for Work Productivity and Activity Impairment (WPAI) questionnaire at 3 and 12 months after enrollment in the digital program, stratified by baseline pain intensity

	All	Mild baseline pain	Severe baseline pain	Difference
*3-month responses*	*N* = 14,607	*N* = 5535	*N* = 9072	
PASS				
WPAI–overall work impairment ^a^	16 (15, 16)	10 (9, 10)	20 (19, 21)	− 10 (− 11, − 9)
WPAI–activity impairment	26 (26, 26)	19 (19, 20)	31 (30, 31)	− 12 (− 12, − 11)
TF				
WPAI–overall work impairment ^a^	29 (28, 31)	18 (16, 22)	34 (32, 36)	− 15 (− 19, − 12)
WPAI–activity impairment	50 (49, 51)	36 (34, 38)	54 (53, 56)	− 19 (− 21, − 16)
*12-month responses*	*N* = 2707	*N* = 1101	*N* = 1606	
PASS				
WPAI–overall work impairment^b^	16 (14, 17)	11 (9, 12)	18 (16, 20)	− 7 (− 10, − 5)
WPAI–activity impairment	26 (25, 27)	21 (20, 22)	29 (28, 30)	− 8 (− 10, − 7)
TF				
WPAI–overall work impairment^b^	33 (29, 39)	21 (16, 27)	40 (33, 47)	− 19 (− 28, − 10)
WPAI–activity impairment	49 (46, 52)	38 (32, 43)	54 (50, 58)	− 16 (− 23, − 9)

^a^The sample sizes were 5564 (All), 2104 (mild), and 3460 (severe)

^b^The sample sizes were 933 (All), 366 (mild), and 567 (severe)

Our subgroup analysis showed that the PASS/TF thresholds of EQ-5D-5L for participants with severe pain were 0.03 to 0.04/0.10 to 0.15 units lower than those with mild pain at baseline (Table 3). For WPAI–work impairment, participants with severe pain had 8–10/15–19 points higher PASS/TF thresholds compared with those with mild pain. Corresponding differences for the WPAI–activity impairment ranged between 8 and 12 for the PASS thresholds and between 16 and 19 for the TF thresholds. Our complete cases analysis (*n* = 2681) showed that 40.5 and 48.2% of participants reported their current state as satisfactory at 3- and 12-month follow-ups, while 2.8% considered the treatment had failed at both follow-ups. We obtained almost identical PASS/TF thresholds among those with complete responses (Table A1 in appendix). Comparing knee vs. hip OA subgroups suggested that while the PASS thresholds were generally comparable between two groups, there were differences in the TF thresholds where hip OA patients tended to consider poorer PROMs as acceptable (Tables A2 and A3 in appendix). The TF/PASS thresholds were comparable for individuals aged ≤ 65 years and those older than 65 years (Table A4 in appendix).

## Discussion

In this study, we estimated the PASS and TF thresholds for EQ-5D-5L and WPAI among a large cohort of persons with knee or hip OA participating in a digital self-management program. Our results showed that at 3 and 12 months following participation in the digital program, around 42–48% of participants considered their current state as satisfactory, while 2–4% considered the treatment had failed. The PASS thresholds for EQ-5D-5L ranged between 0.87 and 0.92 and the TF thresholds ranged between 0.75 and 0.77, with higher PASS thresholds for the Swedish hypothetical than experience-based value set. The estimated PASS and TF thresholds for WPAI were 16–26 and 29–50, respectively, with higher thresholds for WPAI–activity than WPAI–work impairments. While we failed to detect any difference in our estimates across follow-up time, the baseline pain severity had significant effects on the estimated thresholds with those with more severe pain at baseline being prepared to accept poorer PROMs.

To our knowledge, only one previous study reported PASS thresholds, ranging from 0.68 to 0.85 for EQ-5D-5L among people undergoing total hip or knee replacement in Canada [20]. Consistent with our finding they also reported variation in the PASS thresholds according to the EQ-5D-5L value set. In the present study, the Swedish hypothetical value set was associated with 0.04- to 0.05-unit higher PASS thresholds compared with the experience-based EQ-5D-5L value set. This is in contrast with the results from Cooper et al. [32] reporting higher PASS thresholds for the Swedish experience-based than the UK hypothetical EQ-5D-3L value set among persons with chronic arthritic diseases. While, the narrower range and higher mean EQ-5D scores for experience based compared with hypothetical-based value sets are well-documented [33–35], the mean Swedish experience-based EQ-5D-5L values are lower than the Swedish hypothetical-based values for mild health states [25]. Given that the most participants in our sample reported no to moderate problems across all dimensions of EQ-5D-5L, the mean hypothetical values were higher than the experience-based values. For instance, at 3-month follow-up, the proportions of participants with no to moderate problems ranged from 92.7% for pain/discomfort to 99.3% for self-care which resulted in 13,234 (90.6%) of participants being in a health state with larger hypothetical than experience-based EQ-5D-5L scores.

For WPAI, our results suggest that participants consider work and activity impairment scores less than 16 and 26 (out of 100), respectively, as acceptable while scores above 29–33 and 49–50 would be considered as treatment failure. Larger thresholds values for WPAI–activity than WPAI–work impairment is possibly due to higher level of activity impairment than work impairment in our sample. For instance, among the participants with both WPAI–activity and –work impairments responses at baseline (*n* = 5453), the mean scores were 37.3% and 24.3%, respectively.

For both PROMs, we found that the estimated thresholds were stable over time. Previous studies have reported mixed findings on the time dependency of the thresholds [17, 18, 20]. Consistent with our finding, Connelly et al. [18] and Giesinger et al. [36] reported time-constant thresholds for EQ-5D-3L. Conner‑Spady et al. [20] reported time-dependent PASS thresholds for EQ-5D-5L among people undergoing total knee replacement when using Canadian value set, while time-constant thresholds were reported when the EQ-5D-5L scores were calculated using crosswalk. Naal et al. [17] reported time-dependent PASS thresholds for EQ-5D-3L among persons with total hip arthroplasty and time-constant PASS threshold among those with total knee arthroplasty.

Our results showed that persons with more severe pain at baseline were willing to accept poorer (more severe) PROMs after participation in the digital program. While the baseline dependency of PASS/TF thresholds for EQ-5D-5L and WPAI has not previously been explored, the baseline dependency of PASS/TF thresholds for other PROMs is well documented [2, 9, 37]. This baseline dependency calls for considering the comparability of population’s characteristics when using the PASS/TF thresholds reported in the present study in other populations. In other words, while the estimated PASS/TF thresholds in the total sample can be applied in the population with similar baseline characteristics (e.g., NRS pain 5, the Swedish hypothetical/experience-based EQ-5D-5L score 0.84/0.82 and work/activity impairments 24/39), for populations with milder/more severe symptoms, the threshold values from our subgroup analysis should be used. Albeit, other factors such as type of intervention and co-existing conditions might also influence the thresholds and hence application in other population should be done with caution.

The observed tendency among participants with hip OA, compared to knee OA, to accept poorer PROMs might be due to a higher proportion of respondents considering the treatment failed in the former group (4.9% vs. 3.8% at 3 months and 3.9% vs. 2.2% at 12 months). Moreover, participants with hip OA who considered the treatment failed had poorer baseline health status than their counterparts with knee OA. For instance, while the EQ-5D-5L scores were comparable for participants with knee and hip OA who responded “no” to the treatment failure question (0.83 vs. 0.82 for the experience-based values and 0.85 vs. 0.84 for the hypothetical values), there were larger differences among those who considered the treatment failed (0.78 vs 0.73 for the experience-based values and 0.77 vs. 0.72 for the hypothetical values). Limited variations in the estimated thresholds across age groups were consistent with previous results on the EQ-5D index score PASS thresholds [16, 19]. This finding implies that the estimated PASS/TF thresholds can be applied across different age subgroups.

Estimating the first TF threshold for EQ-5D-5L and first TF/PASS thresholds for WPAI, a large sample of persons with knee or hip OA participating in a digital first-line treatment, and the use of a less biased and more precise approach to estimate the thresholds are the main strengths of the present study. Using a dichotomized anchor question reflecting patients’ own judgment on satisfaction with their symptoms was another strength of the current study [18]. However, several limitations of the study should be considered when interpreting the findings. While the correlation between anchor questions and PROMs were generally acceptable confirming their validity, the anchor-PROM correlation for the WPAI–work activity and TF anchor question at 3-month follow-up was inadequate (< 0.30) [38] which calls for caution in interpreting this threshold. The rate of satisfaction relies on the focus of the anchor question [39]. In the present study, the PASS/TF anchor questions focused on participants’ satisfaction with their current state of knee/hip symptoms and functions which are different from the focus of EQ-5D-5L (overall health-related quality of life) and WPAI (work and activity). This might have influenced the correlations and accuracy of our estimates, particularly for work impairment which has less overlap with symptoms/functions than health-related quality of life and daily activity. Although the anchor questions used in the current study are widely used to estimate PASS/TF thresholds [6, 8, 10], there is no gold standard to capture patients’ satisfaction and alternative anchor questions with different wording might result in different thresholds. The study cohort included individuals with self-reported OA who self-selected to participate in a digital program. These individuals are different from those participating in the face-to-face OA core treatment [40] as well as from those identified in routine practice [41], particularly with higher proportion of females and high educated people in the digital program. Most individuals (91%) participated in the program during the COVID-19 pandemic which could influence their health status and responses to the anchor questions and RROMs, especially 3993 (24%) individuals participating during February–December 2020 prior to initiation of COVID-19 vaccination in Sweden and hence limit the generalizability of our findings. There were some differences between those included in the analysis and those excluded because of missing responses. These might limit the generalizability of our findings. WPAI captures work impairments among people who are employed and hence the findings are not applicable to individuals who lost their jobs due to OA.

## Conclusion

This study provides the first PASS and TF thresholds for EQ-5D-5L and WPAI among persons undergoing a digital first-line treatment for OA. These thresholds might facilitate meaningful interpretation of these PROMs among people with knee or hip OA. Our results suggest that the EQ-5D-5L and WPAI PASS/TF thresholds were stable over time and hence can be applied across different time points after first-line treatments for OA. However, observed variations by value set (for EQ-5D-5L) and baseline pain intensity might limit their generalizability and hence should be applied with great caution in other settings/populations.

### Supplementary Information

Below is the link to the electronic supplementary material.

Supplementary file1 (PDF 642 KB)

## Data Availability

The data that support the findings of this study are available from Joint Academy® but restrictions apply to the availability of these data, which were used under ethical permission for the current study and so are not publicly available. Data may be made available through the corresponding author upon reasonable request and with permission of Joint Academy®.
