# Supplementary material for: Patient acceptable symptom state and treatment failure threshold values for work productivity and activity Impairment and EQ-5D-5L in osteoarthritis
Source: Qual Life Res. 2024 Feb 26;33(5):1257–66. doi: 10.1007/s11136-024-03602-6 (PMC11045603; doi:10.1007/s11136-024-03602-6)
Supplement: Supplementary file 1 — Supplementary file1 (PDF 642 KB) [file 11136_2024_3602_MOESM1_ESM.pdf]

Title: Patient acceptable symptom state and treatment failure threshold values for work productivity and activity Impairment and EQ-5D-5L in osteoarthritis

Journal: Quality of Life Research

Authors: Ali Kiadaliri, Anna Cronström, Leif E Dahlberg, L Stefan Lohmander

Correspondence to: Ali Kiadaliri, Skåne University Hospital, Clinical Epidemiology Unit, Remissgatan 4, SE-221 85 Lund, Sweden (ali.kiadaliri@med.lu.se).

Table A1. Patient acceptable symptom state (PASS) and treatment failure (TF) thresholds (95% confidence intervals) for EQ-5D-5L and Work Productivity and Activity Impairment (WPAI) among participants with responses at both 3- and 12-month follow ups (n=2,681).

	PASS		TF	
	3-Month	12-month	3-Month	12-month
Hypothetical-based EQ-5D-5L score	0.92 (0.92, 0.92)	0.91 (0.91, 0.92)	0.79 (0.77, 0.81)	0.75 (0.73, 0.77)
Experience-based EQ-5D-5L score	0.88 (0.87, 0.88)	0.87 (0.86, 0.87)	0.77 (0.76, 0.79)	0.75 (0.73, 0.77)
WPAI– overall work impairment [a]	16 (14, 17)	16 (14, 17)	31 (26, 35)	33 (29, 39)
WPAI–activity impairment	25 (24, 26)	26 (25, 27)	48 (45, 51)	49 (45, 52)

[a] Employed participants aged 70 years and younger at enrolment (n=925).

Table A2. Patient acceptable symptom state (PASS) and treatment failure (TF) thresholds (95% confidence intervals) for EQ-5D-5L at 3- and 12-month after enrolment in the digital program, stratified by osteoarthritis site.

	PASS			TF		
	Knee OA	Hip OA	Difference (knee-hip)	Knee OA	Hip OA	Difference (knee-hip)
**3-Month**	**N=8756**	**N=5851**		**N=8756**	**N=5851**	
Hypothetical-based EQ-5D-5L score	0.92 (0.92, 0.92)	0.91 (0.91, 0.91)	0.01 (0.01, 0.02)	0.79 (0.78, 0.80)	0.74 (0.72, 0.75)	0.05 (0.04, 0.07)
Experience-based EQ-5D-5L score	0.88 (0.88, 0.88)	0.87 (0.86, 0.87)	0.01 (0.01, 0.02)	0.78 (0.78, 0.79)	0.74 (0.73, 0.75)	0.04 (0.03, 0.05)
**12-month**	**N=1677**	**N=1030**		**N=1677**	**N=1030**	
Hypothetical-based EQ-5D-5L score	0.91 (0.91, 0.92)	0.91 (0.90, 0.92)	0.00 (-0.01, 0.01)	0.78 (0.75, 0.81)	0.72 (0.67, 0.75)	0.06 (0.02, 0.11)
Experience-based EQ-5D-5L score	0.87 (0.87, 0.88)	0.86 (0.86, 0.87)	0.01 (0.00, 0.02)	0.78 (0.75, 0.80)	0.73 (0.70, 0.75)	0.05 (0.02, 0.08)

Table A3. Patient acceptable symptom state (PASS) and treatment failure (TF) thresholds (95% confidence intervals) for Work Productivity and Activity Impairment (WPAI) at 3- and 12-month after enrolment in the digital program, stratified by osteoarthritis site.

	PASS			TF		
	Knee OA	Hip OA	Difference (knee-hip)	Knee OA	Hip OA	Difference (knee-hip)
**3-Month**	**N=8756**	**N=5851**		**N=8756**	**N=5851**	
WPAI– overall work impairment [a]	16 (15, 17)	15 (14, 16)	0.6 (-0.6, 1.7)	29 (27, 32)	29 (27, 32)	0.3 (-3.4, 3.9)
WPAI–activity impairment	26 (26, 27)	26 (25, 26)	0.5 (-0.2, 1.3)	48 (47,50)	51 (49, 53)	-2.6 (-4.8, -0.4)
**12-month**	**N=1677**	**N=1030**		**N=1677**	**N=1030**	
WPAI– overall work impairment [b]	16 (14, 18)	15 (13, 17)	0.8 (-1.9, 3.5)	35 (27, 42)	32 (25, 40)	2.4 (-8.2, 13.4)
WPAI–activity impairment	27 (26, 28)	25 (24, 26)	1.6 (-0.1, 3.4)	45 (40, 50)	52 (48, 57)	-7.0 (-13.1, -0.2)

[a] Employed participants aged 70 years and younger at enrolment (n=3,409 for knee OA and n=2,155 for hip OA).

[b] Employed participants aged 70 years and younger at enrolment (n=574 for knee OA and n=359 for hip OA).

Table A4. Patient acceptable symptom state (PASS) and treatment failure (TF) thresholds (95% confidence intervals) for EQ-5D-5L and Work Productivity and Activity Impairment (WPAI) at 3- and 12-month after enrolment in the digital program, stratified by age group.

	PASS			TF		
	≤65 years	>65 years	Difference (older-younger)	≤65 years	>65 years	Difference (older-younger)
**3-Month**	**N=7929**	**N=6678**		**N=7929**	**N=6678**	
Hypothetical-based EQ-5D-5L score	0.91 (0.91, 0.92)	0.92 (0.92, 0.92)	0.01 (0.00, 0.01)	0.76 (0.75, 0.77)	0.78 (0.77, 0.79)	0.02 (0.01, 0.04)
Experience-based EQ-5D-5L score	0.87 (0.87, 0.87)	0.88 (0.87, 0.88)	0.00 (0.00, 0.01)	0.76 (0.75, 0.77)	0.77 (0.76, 0.78)	0.01 (0.00, 0.02)
WPAI–activity impairment	25 (25, 26)	27 (26, 27)	1.7 (1.0, 2.4)	49 (47, 50)	51 (50, 53)	2.5 (0.2, 4.8)
**12-month**	**N=1434**	**N=1273**		**N=1434**	**N=1273**	
Hypothetical-based EQ-5D-5L score	0.91 (0.90, 0.92)	0.91 (0.91, 0.92)	0.01 (-0.00, 0.02)	0.75 (0.72, 0.78)	0.74 (0.70, 0.78)	-0.01 (-0.06, 0.05)
Experience-based EQ-5D-5L score	0.87 (0.86, 0.87)	0.87 (0.86, 0.88)	0.00 (-0.00, 0.01)	0.75 (0.73, 0.77)	0.75 (0.72, 0.77)	0.00 (-0.03, 0.03)
WPAI–activity impairment	25 (24, 26)	27 (26, 29)	2.5 (0.9, 4.1)	47 (43, 52)	51 (46, 56)	3.9 (-2.2, 10.2)
